# Combined Immune Therapy for the Treatment of Visceral Leishmaniasis

**DOI:** 10.1371/journal.pntd.0004415

**Published:** 2016-02-12

**Authors:** Rebecca J. Faleiro, Rajiv Kumar, Patrick T. Bunn, Neetu Singh, Shashi Bhushan Chauhan, Meru Sheel, Fiona H. Amante, Marcela Montes de Oca, Chelsea L. Edwards, Susanna S. Ng, Shannon E. Best, Ashraful Haque, Lynette Beattie, Louise M. Hafner, David Sacks, Susanne Nylen, Shyam Sundar, Christian R. Engwerda

**Affiliations:** 1 QIMR Berghofer Medical Research Institute, Brisbane, Australia; 2 Queensland University of Technology, Institute of Health and Biomedical Innovation, Brisbane, Australia; 3 Netaji Subhas Institute of Technology, New Delhi, India; 4 Banaras Hindu University Institute of Medical Sciences, Varanasi, Uttar Pradesh, India; 5 Griffith University, Institute of Glycomics, Gold Coast, Australia; 6 University of Queensland, School of Medicine, Brisbane, Australia; 7 Griffith University, School of Natural Sciences, Nathan, Australia; 8 National Institute of Allergy and Infectious Diseases, National Institutes of Health, Bethesda, Maryland; 9 Karolinska Institute, Stockholm, Sweden; University of Notre Dame, UNITED STATES

## Abstract

Chronic disease caused by infections, cancer or autoimmunity can result in profound immune suppression. Immunoregulatory networks are established to prevent tissue damage caused by inflammation. Although these immune checkpoints preserve tissue function, they allow pathogens and tumors to persist, and even expand. Immune checkpoint blockade has recently been successfully employed to treat cancer. This strategy modulates immunoregulatory mechanisms to allow host immune cells to kill or control tumors. However, the utility of this approach for controlling established infections has not been extensively investigated. Here, we examined the potential of modulating glucocorticoid-induced TNF receptor-related protein (GITR) on T cells to improve anti-parasitic immunity in blood and spleen tissue from visceral leishmaniasis (VL) patients infected with *Leishmania donovani*. We found little effect on parasite growth or parasite-specific IFNγ production. However, this treatment reversed the improved anti-parasitic immunity achieved by IL-10 signaling blockade. Further investigations using an experimental VL model caused by infection of C57BL/6 mice with *L*. *donovani* revealed that this negative effect was prominent in the liver, dependent on parasite burden and associated with an accumulation of Th1 cells expressing high levels of KLRG-1. Nevertheless, combined anti-IL-10 and anti-GITR mAb treatment could improve anti-parasitic immunity when used with sub-optimal doses of anti-parasitic drug. However, additional studies with VL patient samples indicated that targeting GITR had no overall benefit over IL-10 signaling blockade alone at improving anti-parasitic immune responses, even with drug treatment cover. These findings identify several important factors that influence the effectiveness of immune modulation, including parasite burden, target tissue and the use of anti-parasitic drug. Critically, these results also highlight potential negative effects of combining different immune modulation strategies.

## Author Summary

Despite a decline in the numbers of visceral leishmaniasis (VL) cases and the availability of drugs to treat patients, there is still a need for more efficient treatment options. This is especially important as many VL endemic areas enter phases of disease elimination where parasite burdens need to be reduced as far as possible to minimize the risk of future parasite transmission. Immunotherapy involving the activation of anti-parasitic immunity in infected individuals is a promising approach to help achieve these goals. We tested whether immune modulation could improve anti-parasitic immunity using VL patient samples and an experimental mouse model of infection. Our results provide guidance on the utility of the experimental model, as well as highlighting potential problems associated with immune modulation to improve disease outcomes. These findings have implications for understanding how immunotherapy should be tested and implemented.

## Introduction

Immune check point inhibitors show great potential for the treatment of various cancers[1,2]. These drugs modulate regulatory pathways which suppress anti-tumour immune responses. Major targets for blockade include CTLA4, PD1, PDL1, LAG3, TIM3 and IL-10, but a number of molecules are also being tested for immune activation, including CD27, CD40, OX40, CD137 and glucocorticoid-induced TNF receptor-related protein (GITR)[3–5]. Many of these molecules play important immunoregulatory roles during infectious diseases, including malaria and leishmaniasis[6,7], thereby providing opportunities to use these treatment strategies in different clinical settings. However, testing immune therapies for treating parasitic diseases lags way behind their development for cancer treatment.

Visceral leishmaniasis (VL) is a potentially fatal disease caused by *Leishmania donovani* in the Indian subcontinent and East Africa, and *L*. *infantum* (*chagasi*) around the Mediterranean and in Central and South America[8]. These protozoan parasites infect macrophages in visceral organs, with the spleen, bone marrow, lymph nodes and liver being the main tissues affected[9,10]. Despite the majority of infections being asymptomatic, individuals that develop VL have a high likelihood of death if they do not receive appropriate chemotherapy[11–13]. Treatment in the Indian subcontinent has improved dramatically with the recent implementation of a single dose administration of liposomal amphotericin B (Ambisome)[14–16]. However, problems remain with this regime, including cost and toxicity[8]. In addition, treatment in other endemic regions still relies on more extensive regimes, such as an up to 30 day course of pentavalent antimonials for VL patients in East Africa[8]. Again, toxicity is an important problem and there is a high chance of relapse and/or development of complications such as post kala-azar dermal leishmaniasis (PKDL)[8]. Therefore, there is an urgent need to improve current treatments.

Interleukin 10 (IL-10) has emerged as a major regulatory cytokine in experimental VL models and in VL patients[7,10,17,18]. IL-10 can act directly on antigen presenting cells to suppress their functions, as well as on T cells to inhibit activity[6,19]. Blockade of IL-10 in whole blood samples from VL patients caused significantly greater IFNγ and TNF production by CD4^+^ T cells in response to stimulation with parasite antigen, thus identifying IL-10 as a potent suppressor of cell-mediated immunity in VL patients[17,20]. However, responses to IL-10 signaling blockade were variable and not always positive, suggesting a level of heterogeneity amongst individuals in the importance of IL-10 for suppressing anti-parasitic immune responses[17]. Other immunoregulatory molecules that influence T cell responses during experimental VL have been identified, including PD1[21], CTLA4[22], OX40[23], CD40[24–26], IL-27[27] and TGFβ[28]. We previously showed that activation of T cells via GITR could enhance anti-parasitic CD4^+^ T cell responses in experimental VL[29]. GITR activation with an agonist mAb improved anti-parasitic CD4^+^ T cell responses, but only when administered after establishment of infection when GITR expression increased on all T cell populations. Furthermore, despite only having a minor effect on established hepatic infection when administered alone, anti-GITR mAb acted synergistically with a sub-optimal dose of anti-parasitic drug to improve parasite clearance in the liver and spleen[29].

Here we extend these findings into a clinical setting by testing whether GITR activation improved antigen-specific immune responses in VL patient samples. We also used patient samples and an experimental model of VL to investigate how GITR activation influenced cellular immune responses during infection when administered alone or in combination with IL-10 signaling blockade. Our results uncover a complex outcome following immune activation in both VL patient samples and experimental VL that is influenced by parasite load and with the use of anti-parasitic drug. These findings have implications for the development of immune therapy to treat infectious diseases, as well as broader implications for understanding potential consequences of this approach in any disease setting.

## Methods

### Ethics statement

All patients presented with symptoms of VL at the Kala-azar Medical Research Center (KAMRC), Muzaffarpur, Bihar, India. Their diagnosis was confirmed either by the detection of amastigotes in splenic aspirate smears or by rk39 dipstick test. Patients were treated either with Amphotericin B or Ambisome. In total, 58 patients and 10 healthy controls were enrolled in this study. The use of human subjects followed recommendations outlined in the Helsinki declaration. Written informed consent was obtained from all participants and/or their legal guardian when under 18 years of age. Ethical approval (Dean/2011-12/289) was obtained from the ethical review board of Banaras Hindu University (BHU), Varanasi, India. The aggregate clinical data of enrolled subjects are given in Table 1.

**Table 1:** Clinical characteristics of VL patients enrolled in this study.

	VL patients	Endemic controls
**Number**	58	10
**Sex (M/F)**	34/24	5/5
**Age (years): Mean ± SD**	30.96±18.15 (28)^a^	36.8 ±13.3 (35)
**Duration of illness (days): Mean ± SD**	52.18± 58.71 (30)	N/A^b^
**WBC count–pre-treatment: Mean ± SD**	4115±1828 (3750)	N/A
**WBC count–post-treatment**^**c**^**: Mean ± SD**	8200±2947 (8200)	N/A
**Spleen size (cm)–pre-treatment: Mean ± SD**	4.2±2.85 (4)	N/A
**Spleen size (cm)–post-treatment**^**c**^**: Mean ± SD**	0.94±1.56 (0)	N/A
**Splenic score**^**d**^**: Mean ± SD**	2.24±1.33(2)	N/A

^a^Values in parentheses represent median values.

^b^N/A = not applicable.

^c^Post-treatment values are from 15 or 30 days after commencement of drug treatment.

^d^Scoring of parasite load is on a logarithmic scale where 0 is no parasites per 1000 microscopic fields (x1000), 1 is 1–10 parasites per 1000 microscopic fields, and 6 is > 100 parasites per 1000 microscopic fields. Splenic scores were only calculated for patients that underwent splenic biopsy. Some patients were treated based on results from the rk39 strip test result.

All animal procedures were approved by the QIMR Berghofer Medical Research Animal Ethics Committee. This work was conducted under QIMR Berghofer animal ethics approval number A02-634M, in accordance with the “Australian Code of Practice for the Care and Use of Animals for Scientific Purposes” (Australian National Health and Medical Research Council).

### Peripheral Blood Mononuclear Cells (PBMC) isolation, real time PCR and surface staining

Heparinised blood was collected from VL patients (n = 7) before and after treatment, as well as from endemic controls (EC; n = 5). PBMCs were isolated by Ficoll-Hypaque (GE Healthcare, NJ) gradient centrifugation and collected directly into RNAlater and stored at -70°C until mRNA isolation and analysis. Total RNA was isolated using RNeasy mini kit and Qiashredder homogenizers (Qiagen, Venlo, Netherlands) according to the manufacturer’s protocol. The quality of RNA was assessed by denaturing agarose gel electrophoresis. cDNA synthesis was performed in 20 μL reactions on 0.5–1.0 μg RNA using High-Capacity cDNA Archive kit (Applied Biosystems, Foster City, CA). Real-time PCR was performed on an ABI Prism 7500 sequence detection system (Applied Biosystems) using cDNA-specific FAM–MGB labelled primer/probe for GITR. The relative quantification of products was determined by the number of cycles over 18S mRNA endogenous control required to detect the gene expression of interest. In a separate experiment, PBMC were isolated from VL patients (n = 7) before drug treatment, as well as from EC’s (n = 5), for cell surface staining of GITR and FACS analysis.

### Spleen cell culture

Prior to treatment, splenic needle aspirates were collected from VL patients (n = 15) at KAMRC. The left over splenic aspirate after formation of a smear on a glass slide (to demonstrate the presence of parasite), was collected in 0.8ml RMPI-1640 medium supplemented with 10% fetal bovine serum (FBS), 10mM L-glutamine, 100U/ml penicillin and 100μg/ml streptomycin (Invitrogen, Carlsbad, CA). Spleen samples were transported to Banaras Hindu University for further analysis at a temperature of 14–18°C. Spleen cell culture was performed as reported previously[17]. In brief, a small fraction of spleen cell suspension (150μl) was inoculated in a 96 well plate containing blood agar for base line quantification of parasites by limiting dilution assay. The remaining sample was plated in duplicate (300μl) in 96 well tissue culture plates (Thermo Fisher, Waltham, MA) and incubated for 3 days at 37°C and 5% CO_2_ in presence of either anti-GITR mAb (clone TRX518; 20μg/ml, Tolerx, Cambridge, MA) or isotype control (human IgG1;Sigma, St Louis, MO). TRX518 is a non-depleting, humanized IgG1 anti-human GITR mAb with a heavy chain asparagine 297 substitution to alanine to eliminate N-linked glycolsylation and abrogate Fc region functionality[30], produced under good manufacturing process (GMP) conditions. After 3 days, culture supernatants were removed and replaced by promastigote growth medium (M199), supplemented with 20% FBS, 10mM L-glutamine, 100U/ml penicillin and 100μg/ml streptomycin, 40mM HEPES, 0.1mM adenine (in 50 mM HEPES), 5mg/ml hemin (in 50% triethanolamine). The splenic aspirates were transferred to blood agar plate for limiting dilution assay using 3-fold serial dilutions and kept at 25°C for 10–12 days to allow parasite growth.

### Whole blood assay

Whole blood assays were performed as previously described[20,31]. In brief, heparinised blood was collected (n = 19) from active VL patients. To remove background plasma cytokines, the plasma was replaced with an equal volume of FBS. Whole blood cells were cultured in absence or in presence of soluble leishmania antigen (SLA). To detect whether GITR activation could enhance antigen specific immune response; either alone or in combination with IL-10 signaling blockade, specific mAbs and their isotype controls (IgG2b and IgG1 for anti-IL-10 and anti-GITR mAbs, respectively) were added (each 20μg/ml) along with SLA. In all experiments, a non-stimulated group was included, and although minimal cytokine production was detected in these samples, the levels detected were subtracted from corresponding antigen-stimulated samples. Anti-IL-10 mAb (clone25209) and mouse IgG2b isotype (clone 20116) were purchased from R&D Systems (Abingdon, United Kingdom). Whole blood cultures were kept at 37°C and 5% CO_2_ for 24 hours. Supernatants were collected and IFN-γ levels were measured using an ELISA kit (Biolegend, San Diego, CA), as per manufacturer instructions. To test the effect of drug treatment on responses to antibodies in whole blood assays, a similar experiment was conducted on a separate set of whole blood samples (n = 10), where heparinized blood was collected from active VL patients before the start of treatment and one day after single-dose Ambisome treatment[15].

### Mice

Female C57BL/6J were purchased from the Australian Resource Centre (Canning Vale, Western Australia) and Walter and Eliza Hall Institute for Medical Research (Victoria, Australia), and maintained under conventional conditions at the QIMR Berghofer Medical Research Institute. B6.RAG2^-/-^ mice were bred at the Queensland Institute of Medical Research. Mice used were sex- and age-matched (6–10 weeks) and groups of 5 to 6 mice were used in experiments.

### Parasites and infections

*L*. *donovani* (LV9 strain) were maintained by passage in B6.RAG2^-/-^ mice and amastigotes were isolated from the spleens of chronically infected animals. Mice were infected by injecting 2 x 10^7^ amastigotes i.v. via the lateral tail vein, killed at the times indicated in the text by CO_2_ asphyxiation and bled via cardiac puncture. In experiments examining low dose infections, mice were infected with 5 x 10^6^ amastigotes i.v.. Spleens and perfused livers were removed at times indicated and parasite burdens were determined from Diff-Quik-stained impression smears (Lab Aids, Narrabeen, Australia) and expressed as Leishman-Donovan units (LDU) (the number of amastigotes per 1,000 host nuclei multiplied by the organ weight in grams)[32]. Spleen parasite burden was also determined by limiting-dilution analysis[17]. Hepatic mononuclear cells and splenocytes were isolated as previously described[33].

### Antibodies and drugs for in vivo administration

All hybridomas (DTA-1 (anti-GITR[34]) and 1B1.3a (anti-IL-10R[35])) were grown in 5% FCS, RPMI 1640 containing 10 mmol/l L-glutamine, 200 U/ml penicillin, and 200 mg/ml streptomycin. Purified mAbs were prepared from culture supernatants by protein G column purification (Amersham Biosciences, Uppsala, Sweden), followed by endotoxin removal (Mustang membranes, Pall, East Hills, NY). For *in vivo* stimulation of GITR, mice were injected intraperitoneal (i.p.) with 0.5 mg DTA-1 mAb in 200μl 0.9% sodium chloride (Baxter) per mouse on day 14 post-infection. Anti-IL-10R blocking mAb (1B1.3a) was administered in 0.5mg doses, via i.p. injection in 200μl 0.9% sodium chloride (Baxter, Old Toongabbie, NSW, Australia) per mouse on days 14, 19 and 24 post-infection. Control mice were administered the same quantities of control rat IgG (BioXcell, West Lebanon, NH) at the same time points as the stimulatory or blocking mAbs. The pentavalent antimonial, sodium stibogluconate (Sb^v^; Albert David Ltd, Kolkata, India], was administered at 500mg/kg/day doses in 200μl 0.9% sodium chloride (Baxter) per mouse on days 14 and 21 post-infection and administered i.p.. In multiple-dosing experiments, mice were treated on days 14, 16, 18, 20, 22, 24 and 26 post-infection with Sb^v^ at 50mg/kg/day doses in 200μl 0.9% sodium chloride (Baxter) per mouse and administered i.p., based on drug dosing used by others[36].

### Flow cytometry

Mouse studies: Brilliant Violet 421-conjugated anti–TCRβ chain (H57-597), PerCP-Cy5.5–conjugated anti–TCRβ chain (H57-597), Brilliant Violet 605–conjugated anti-NK1.1 (PK136), Allophycocyanin-Cy7–conjugated anti-NK1.1 (PK136), Brilliant Violet 605–conjugated anti-CD4 (GK1.5), Allophycocyanin-Cy7–conjugated anti-CD4 (GK1.5), Alexa Fluor 700–conjugated anti-CD8α (53–6.7), FITC–conjugated anti-CD11a (M1714), PE-Cy7–conjugated anti-CD49d (R1-2), Biotinylated anti-CD49d (R1-2), PerCP-Cy5.5–conjugated anti–KLRG-1 (2F1), PE-Cy7–conjugated anti-CD25 (PC61), Allophycocyanin-conjugated anti-Foxp3 (MF14), Allophycocyanin-conjugated anti-Tbet (eBio4B10), PE-Cy7-conjugated anti-Tbet (eBio4B10), PE-conjugated anti-IFNγ (XMG1.2), Allophycocyanin-conjugated anti-IFNγ (XMG1.2), Brilliant Violet 421-conjugated anti-IFNγ (XMG1.2), PE-conjugated anti-IL-10 (JES5-16E3), Allophycocyanin-conjugated anti-IL-10 (JES5-16E3), PE-conjugated anti-TNFα (MP6-XT22), PE-conjugated anti-CD279 (PD-1) (J43) and Allophycocyanin-conjugated anti-CD223 (LAG-3) (C9B7W) were purchased from BioLegend or BD Biosciences (Franklin Lakes, NJ). Biotinylated antibodies were detected with streptavidin conjugated PE-Cy7. Dead cells were excluded from the analysis using LIVE/DEAD Fixable Aqua Stain or LIVE/DEAD Fixable Near I-R Stain (Invitrogen-Molecular Probes, Carlsbad, CA), according to the manufacturer’s instructions. The staining of cell surface antigens and intracellular cytokine staining were carried out as described previously[37]. FACS was performed on aLSRFortessa (BD Biosciences), and data were analysed using FlowJo software (TreeStar, Ashland, OR). Serum and/or tissue culture supernatants were assessed for the presence of soluble cytokines using flexset bead array kits (BD Biosciences) according to the manufacturers’ instructions.

Human studies: Allophycocyanin-conjugated anti-CD3ε (HIT3a) and FITC–conjugated anti-CD4 (A161A1) were purchased from Biolegend. PE-conjugated GITR (110416) was purchased from R&D Systems. FACS was performed on a BD FACS Calibur and data were analysed using FlowJo software.

### Complete medium for lymphocyte culture

RPMI 1640 was supplemented with 20% heat in activated FCS, penicillin (2Mm), streptomycin (1000 U/ml), 2-mercaptoethanol (0.05 mM), sodium pyruvate (2 mM) and 1M HEPES (200 μm Final).

### Antigen-specific cellular analysis

Mouse spleens were processed to obtain a single-cell suspension, diluted at 2 x 10^6^ cells/ml in RPMI 1640 complete medium and aliquoted in 96 well plates at 1 x 10^5^ cells/well. Cells were stimulated with fixed *L*. *donovani* amastigotes at 2 x 10^6^ parasites/well for 24 hours, as previously described[38]. In all experiments, 100μL/well supernatant was removed 4 hours prior to cell harvest for measurement of cytokine levels and replaced with 100μL/well fresh RPMI 1640 complete medium containing Brefeldin A (Sigma, St Louis, MO), at a final concentration of 10 μg/ml. Intracellular cytokine staining was performed as described above.

### Statistical analysis

Comparisons between two groups were performed using non-parametric Mann-Whitney tests in mouse studies and Wilcoxon matched-pairs signed rank test or paired t-test, as appropriate, in human studies. Comparisons between multiple groups were made using a Kruskal-Wallis test and corrected using Dunn’s multiple comparisons test. Differences of p< 0.05 were considered significant. Graphs depict mean values ± SEM. All statistical analyses were performed using GraphPad Prism 6 software.

## Results

### GITR expression increases on CD4^+^ T cells during VL, but GITR activation does not improve parasite-specific cellular immune responses

**Fig 1:**
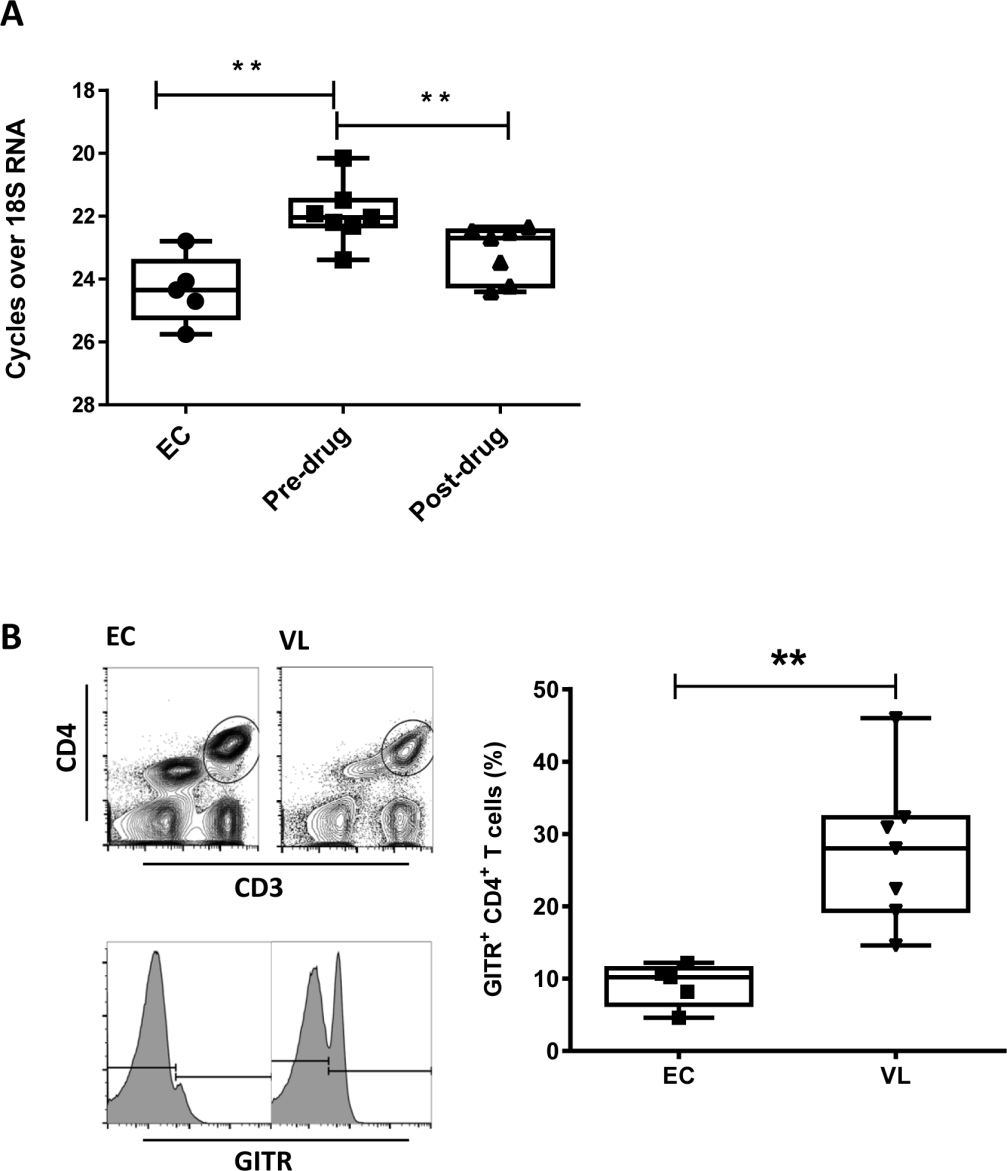
Increased frequency of GITR-positive CD4^+^ T cells in VL patients. **A.** The relative expression of GITR mRNA in PBMC of VL patients was measured by qPCR before treatment (Pre-drug; n = 7) and 28 days after the commencement of treatment (Post-drug; n = 7),as well as in healthy endemic control (EC; n = 5) samples. **B**. PBMC’s from VL patients before drug treatment (VL; n = 7) and healthy endemic controls (EC; n = 5) were gated on CD3ε^+^ CD4^+^ T cells and the frequency of GITR-positive CD4^+^ T cells was measured by FACS. Box and whisker plots show the box extending from the 25^th^ to 75^th^ percentiles with the line in the middle of the box representing the median and whiskers going down to the smallest value and up to the largest. Statistical differences of p < 0.05 (*) and p < 0.01 (**) are indicated.

We first examined the therapeutic potential of GITR in VL patients by measuring GITR mRNA accumulation in PBMC’s from VL patients before and after drug treatment, and comparing with GITR mRNA accumulation in PBMC’s from healthy, endemic controls (Fig 1A). GITR mRNA levels increased in cells from VL patients, compared with endemic control samples, and then declined after the completion of drug treatment. FACS analysis on blood samples revealed that there was a significantly higher frequency of GITR-positive CD4^+^ T cells in VL patients, compared to endemic controls (Fig 1B). Therefore, similar to our findings in experimental VL[29], there was a greater frequency of GITR-positive CD4^+^ T cells during active VL, thus making this molecule a potential therapeutic target.

**Fig 2:**
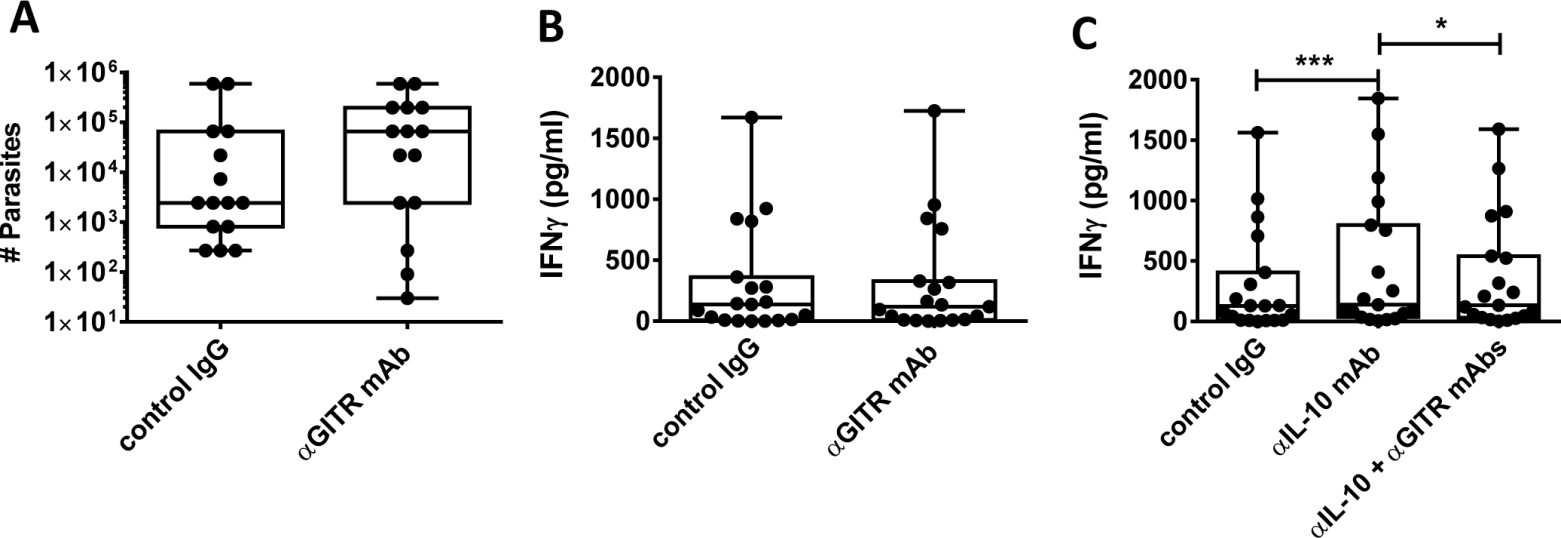
GITR activation has no significant impact on parasite growth in spleen samples and antigen-specific IFN-γ production in whole blood from VL patients. **A.** Spleen cells were cultured in the presence of agonistic anti-GITR mAb or control IgG1, as indicated, before counting the number of viable amastigotes present after 3 days by limiting dilution in blood agar plates (n = 15). **B.** Antigen-specific IFN-γ production was measured in whole blood cell cultures after 24 hours of stimulation with agonistic anti-GITR mAb or control IgG1, as indicated (n = 19). C. Antigen-specific IFN-γ production was measured in whole blood cell cultures after 24 hours of stimulation with a blocking anti-IL-10 mAb, with or without agonistic anti-GITR mAb, and compared with samples treated with control IgG1, as indicated (n = 19). Box and whisker plots show the box extending from the 25^th^ to 75^th^ percentiles with the line in the middle of the box representing the median and whiskers going down to the smallest value and up to the largest. Statistical differences of p < 0.05 (*) and p < 0.001 (***) are indicated.

We next tested whether GITR can be targeted to improve parasite killing by adding anti-GITR mAb to cell cultures comprising splenic aspirates taken from VL patients as part of routine diagnostic procedures. We used a non-depleting, humanized IgG1, anti-human GITR mAb with reported ability to enhance effector T cell responses and block regulatory T cell-mediated suppression[30]. We found no change in the number of viable parasites in culture following anti-GITR mAb addition, compared with control samples (Fig 2A), suggesting that targeting GITR had minimal impact on anti-parasitic immune responses under these conditions. To examine whether targeting GITR had any effects on cell-mediated immune responses in VL patients, we next employed a whole blood assay in which cells were cultured overnight in the presence of parasite antigen and IFNγ levels were measured the following day, as previously reported[17,20]. Initial experiments showed no improvement in antigen-driven IFNγ production following the addition of anti-GITR mAb (Fig 2B). Past work demonstrated that IL-10 was a major suppressor of anti-parasitic immunity in this assay[17], and hence, we speculated IL-10 might prevent any positive effects of targeting anti-parasitic IFNγ production. Therefore, we tested whether combining anti-GITR mAb treatment with IL-10 signaling blockade could improve this response. As previously reported[17,20], IL-10 signaling blockade resulted in increased IFNγ production in this assay following stimulation with parasite antigen (Fig 2C). Surprisingly, the addition of an anti-GITR mAb reversed this effect (Fig 2C). Thus, targeting GITR on antigen-activated PBMC’s from VL patients had no effect alone, and a detrimental effect on their ability to respond to IL-10 signaling blockade.

### GITR activation interferes with the anti-parasitic effects of IL-10 blockade following a low-dose infection

**Fig 3:**
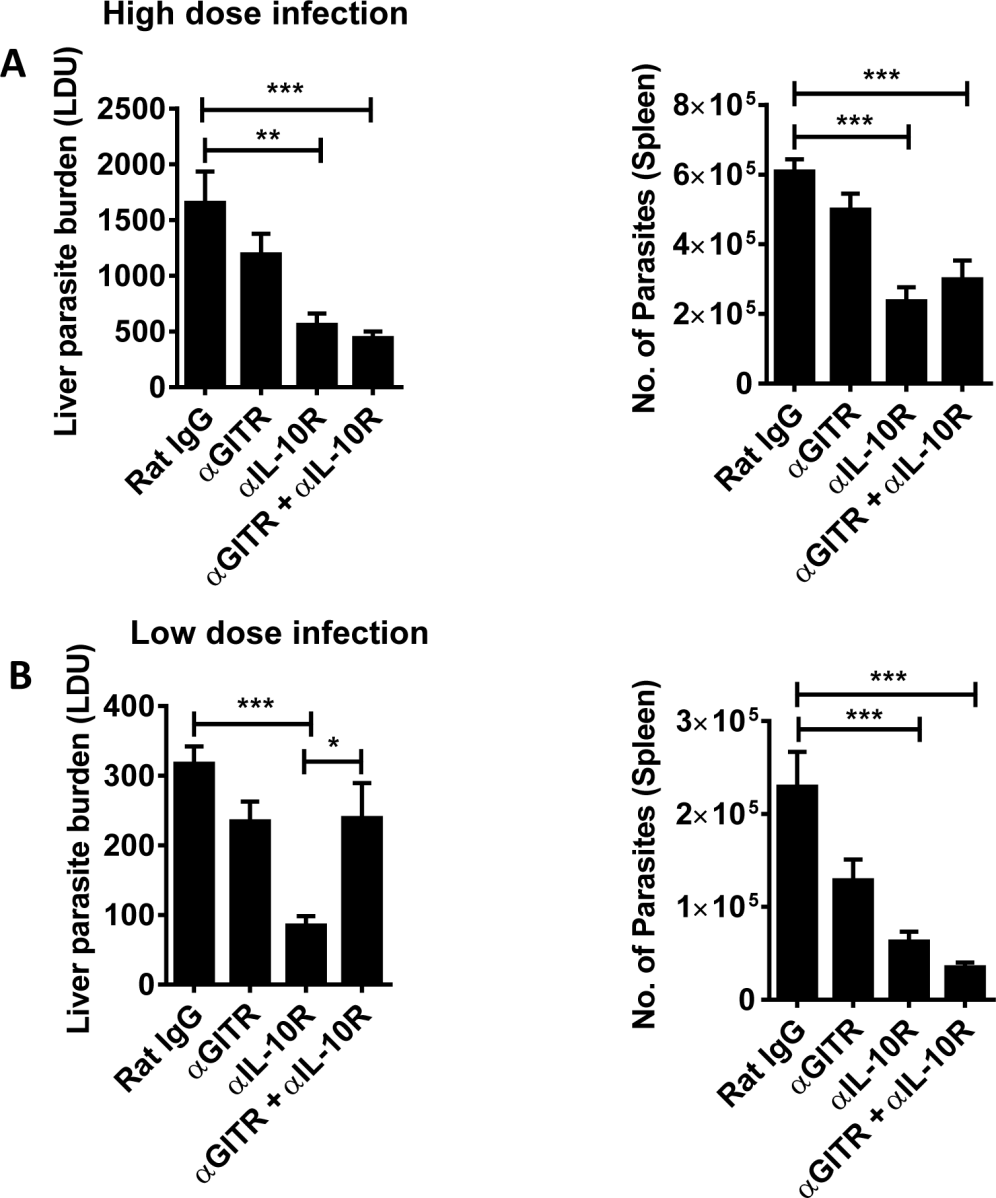
Parasite inoculum determines combination treatment outcome. Parasite burdens at day 28 p.i., were measured in the livers and spleens of mice, as indicated, infected with a high (**A**) or low (**B**) dose of parasite inoculum. Infected mice were treated with mAb alone or a combination of anti-GITR mAb on day 14 p.i., and anti-IL-10R mAb on days 14, 19 and 24 p.i.. Rat IgG was used as a control. Data are represented as the mean +/- SEM, and statistical differences of p < 0.05 (*), p <0.01 (**) and p < 0.001 (***) are indicated (n = 15 mice per group from 3 independent experiments).

To try and better understand the above results, we returned to the experimental VL model caused by intravenous infection of C57BL/6 mice with *L*. *donovani* amastigotes. Infections were allowed to establish for 14 days before treating mice with an agonistic anti-GITR mAb, an anti-IL-10R mAb or a combination of both, and then measuring parasite burdens 14 days later (Fig 3A). Although there was a consistent reduction in parasite burdens following anti-GITR mAb administration, this did not reach statistical significance, relative to control treated mice, in line with our previous findings[29]. As expected[39,40], IL-10 signaling blockade resulted in significantly reduced parasite burdens in both liver and spleen, compared with controls, but combining this treatment with GITR activation had little impact on parasite burdens. Serendipitously, in an experiment where mice were mistakenly infected with a 4-fold reduction in parasite number, we observed that GITR activation limited the anti-parasitic activity caused by IL-10 signaling blockade in the liver, as we had observed in our human studies. Therefore, we established infections using a lower parasite inoculum and examined the impact of immune modulation (Fig 3B). Again, despite a consistent reduction in parasite burdens following anti-GITR mAb administration, this did not reach statistical significance, relative to control treated mice. IL-10 signaling blockade resulted in significantly reduced parasite burdens in both liver and spleen, compared with controls, but combining this treatment with GITR activation limited these effects in the liver. Therefore, the liver response in this lower burden experimental VL model appeared to better reflect the immune environment in human VL patients, so we therefore included the lower parasite burden model in our studies and focused on responses in this tissue site.

### Low-dose infection and immune modulation promotes KLRG-1 expression by Th1 cells

**Fig 4:**
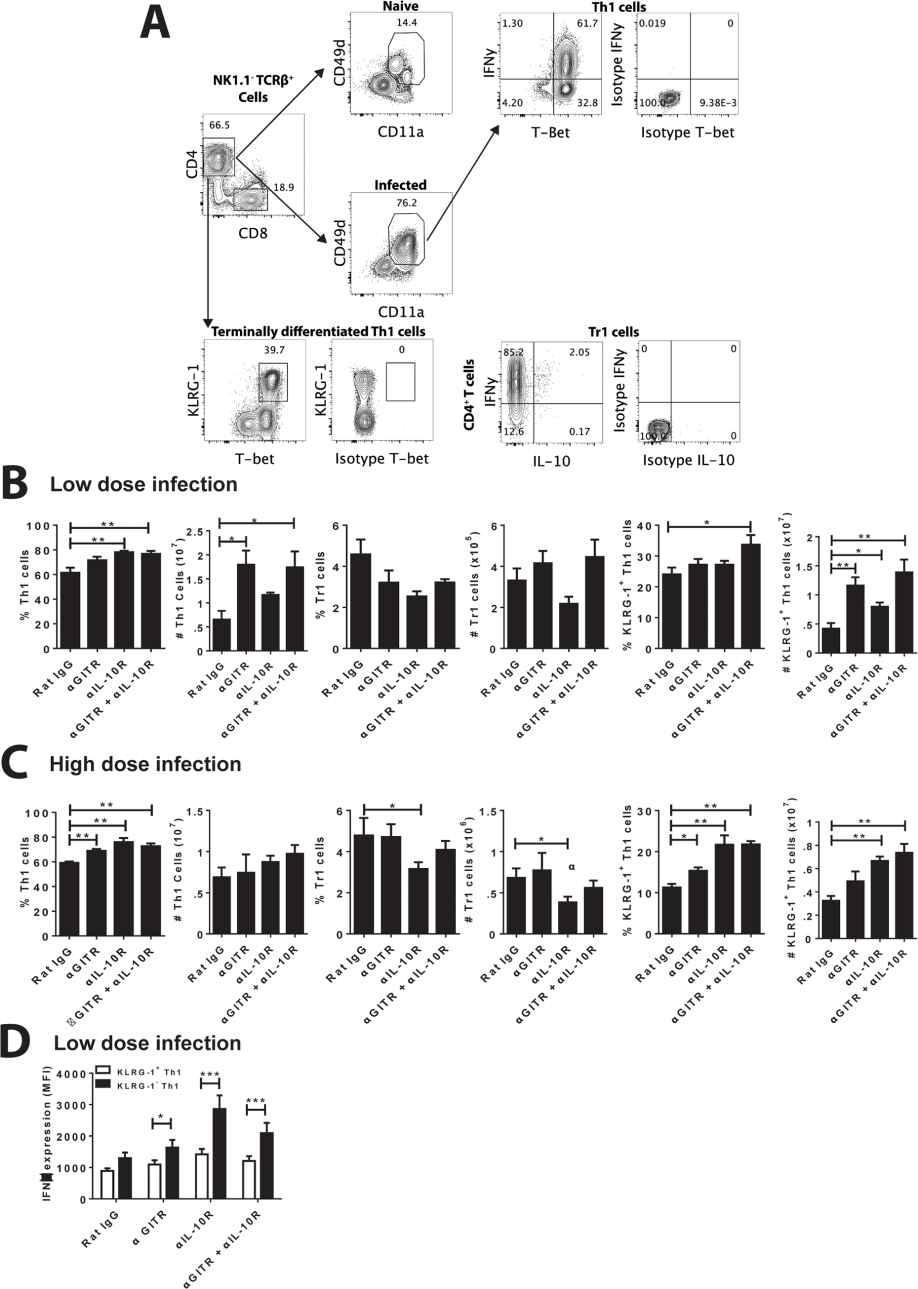
Combination antibody treatment results in increased KLRG-1 expression by Th1 cells in the liver. **A**. Hepatic CD4^+^ T cells from mice infected with a low (**B and D**) and high (**C**) parasite inoculum, and treated as indicated, were analysed by FACS by first gating on CD4^+^ and CD8^+^populations in the TCRβ^+^ NK 1.1^-^ cell fraction. Activated CD4^+^ T cells were selected based on high levels of CD49d and CD11a expression. Intracellular cytokine staining on activated CD4^+^ T cells was used to identify Th1 (Tbet^+^ IFNy producing CD4^+^ T cells; measured directly *ex vivo*), Tr1 (IL-10 and IFNy producing CD4^+^ T cells; measured after 3 hours stimulation *ex vivo* with PMA and ionomycin) and terminally differentiated Th1 (Tbet^+^ KLRG-1^+^ CD4 T cells) cells. Infected mice were treated with the mAb alone or a combination of anti-GITR mAb on day 14 p.i., and anti-IL-10R mAb on days 14, 19 and 24 p.i., as indicated. Rat IgG was used as a control. Both the frequency and total number of cells are shown for each CD4^+^ T cell subset. **D.** The mean fluorescence intensity (MFI) of IFNγ expression on KLRG-1^+^ and KLRG-1^-^ Th1 cells was also measured. Data are represented as the mean +/- SEM at day 28 p.i.. Statistical differences of p < 0.05 (*) and p < 0.01 (**) are indicated (n = 5 mice per group, one representative experiment from 3 independent experiments).

We next examined various immune parameters that might explain the negative impact of GITR activation. Th1 (Tbet^+^, IFNγ-producing CD4^+^ T cells) cells[41] and IL-10-producing CD4^+^ T cells[18] cells have been previously shown to influence disease outcome in experimental and clinical VL, but we found only relatively minor differences in these T cell populations, as well as IL-10-producing Th1 (Tr1) cells, in the liver between mice treated with different combinations of agonistic anti-GITR and anti-IL-10R mAbs, regardless of whether mice were infected with a low (5 x 10^6^; Fig 4B) or high (2 x 10^7^; Fig 4C) parasite inoculum. Similar observations were also made in spleen tissue (S1 Fig). Interestingly, in the spleen we observed a greater number of Th1 cells in control mice with low dose infection, compared to the same group in with high dose infection, but a similar number of Tr1 cells in both groups (S1 Fig). Thus, the ratio of Th1 to Tr1 cells was much higher in the control mice receiving the lower dose of infection.

Differences in both frequency and number of KLRG-1-expressing Th1 cells, possibly representing functionally exhausted cells[42,43], were apparent (Fig 4B and 4C), with significant increases noted in the livers of mice treated with combined anti-GITR and anti-IL-10R mAbs, compared with control mice. However, no significant difference in the frequency or number of KLRG-1-expressing Th1 cells were found in the livers of low dose-infected mice treated with anti-IL-10R mAb alone and mice treated with combined anti-GITR and anti-IL-10R mAbs (Fig 4B), suggesting changes in KLRG-1 expression on Th1 cells may not explain the antagonistic effects of GITR activation on the anti-parasitic effects of IL-10 signaling blockade. Instead, we found that the KLRG-1^+^ CD4^+^ T cells produced significantly more IFNγ on a per cell basis in all antibody-treated groups (Fig 4D). Remarkably, mice infected with lower parasite numbers (Fig 4B) had significant increases in the frequency and number of KLRG-1-expressing Th1 cells, compared with corresponding treatment groups of mice infected with higher parasite numbers (Fig 4C). For example, control mice infected with the low-dose inoculum had 28.2 ± 1.5% and 6.0 x 10^6^ ± 6.1 x 10^5^ KLRG-1^+^ hepatic Th1 cells, compared with 18.0 ± 1.5% and 3.7 x 10^6^ ± 3.6 x 10^5^ KLRG-1^+^ hepatic Th1 cells in the same group infected with the higher dose (P < 0.001 for both frequency and number; n = 16–19 mice/treatment group). Similarly, combined anti-GITR and anti-IL-10R mAb-treated mice infected with the low-dose inoculum had 41.1 ± 1.9% and 9.8 x 10^6^ ± 1.0 x 10^6^ KLRG-1^+^ hepatic Th1 cells, compared with 30.4 ± 2.3% and 7.0 x 10^6^ ± 4.9 x 10^5^ KLRG-1^+^ hepatic Th1 cells in the same group infected with the higher dose (P < 0.01 for frequency and P < 0.001 for number; n = 16–19 mice/treatment group). Again, similar observations were made in the spleen (S1 Fig). Hence, a lower infection dose resulted in more KLRG-1-expressing Th1 cells, but although this was exacerbated following all antibody treatment, it did not appear to explain why GITR activation reversed the anti-parasitic effects of IL-10 signaling blockade.

**Fig 5:**
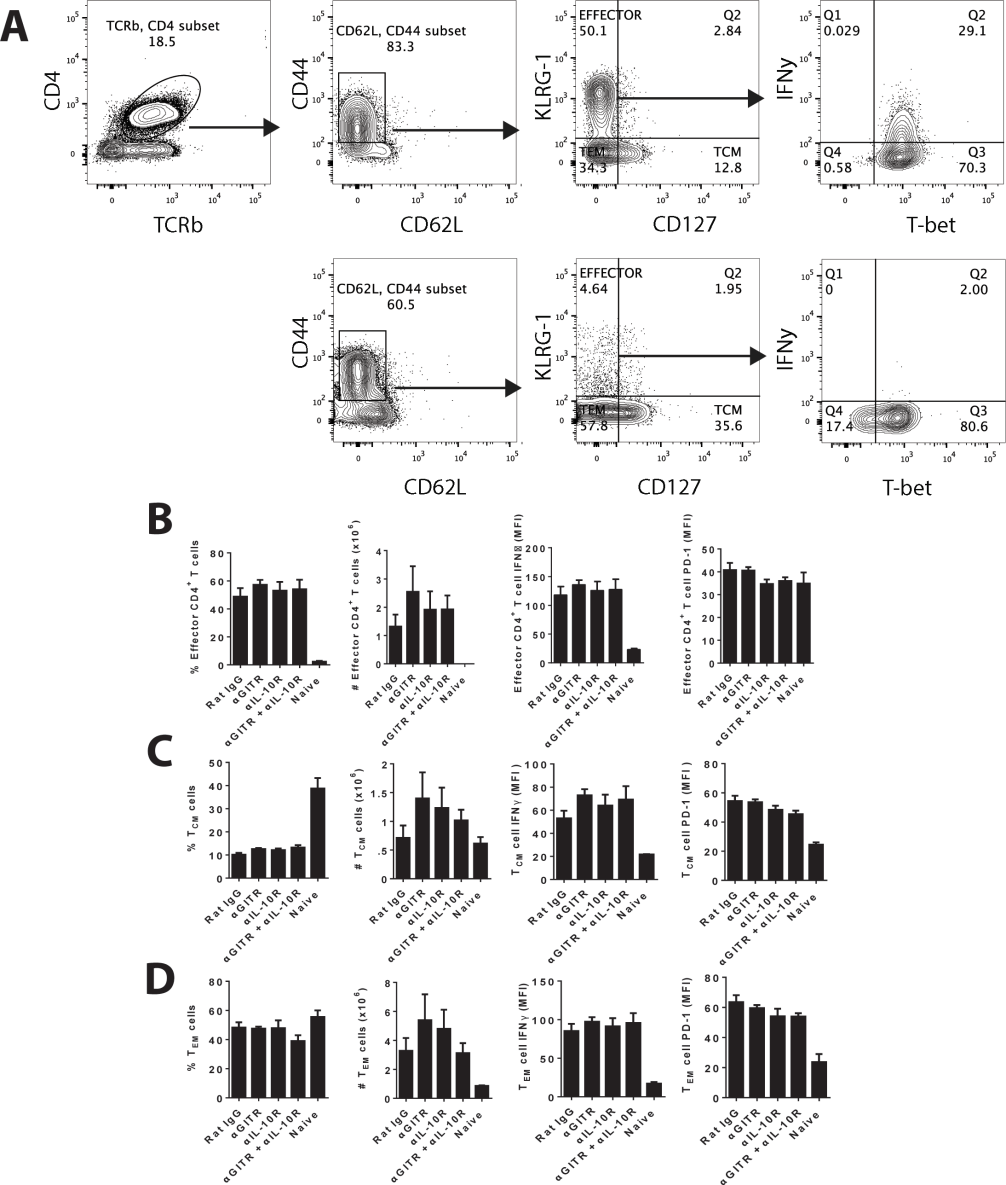
Combination antibody treatment caused little change in effector or memory CD4^+^ T cells in the liver. Mice infected with a low (5 x 10^6^) parasite inoculum, and treated with mAb alone or a combination of anti-GITR mAb on day 14 p.i., and anti-IL-10R mAb on days 14, 19 and 24 p.i., as indicated. Rat IgG was used as a control. **A**. Hepatic CD4^+^ T cells were analysed by FACS by first gating on CD4^+^ TCRβ^+^ populations. Activated CD4^+^ T cells were selected based on CD44 and CD62L expression, and then further divided based on KLRG-1 and CD127 expression. **B.** Effector CD4^+^ T cells were defined as KLRG-1^+^ CD127^-^, **C.** central memory T (T_CM_) CD4^+^ T cells were defined as KLRG-1^-^ CD127^+^, and **D.** effector memory T (T_EM_) CD4^+^ T cells were defined as KLRG-1^-^ CD127^-^) frequency and number are shown (n = 5 mice per group, one independent experiment from 1–3 independent experiments).

Additional analysis of the frequency or number of hepatic effector (Fig 5B), central memory (T_CM_; Fig 5C) and effector memory (T_EM_; Fig 5D) CD4^+^ T cells, based on previously described markers[44,45], revealed few differences in any of the treated groups. Furthermore, the expression of IFNγ and PD-1 on these CD4^+^ T cell subsets was not different between infected groups (Fig 5B–5D). Thus, it is unlikely that the negative impact of anti-GITR mAb treatment on IL-10 signaling blockade can be explained by either the promotion of T cell exhaustion or impairment of T cell activation or memory T cell differentiation.

### Combining immune modulation with drug treatment improved anti-parasitic immunity, but IL-10 signaling blockade alone is better than combining with targeting GITR

**Fig 6:**
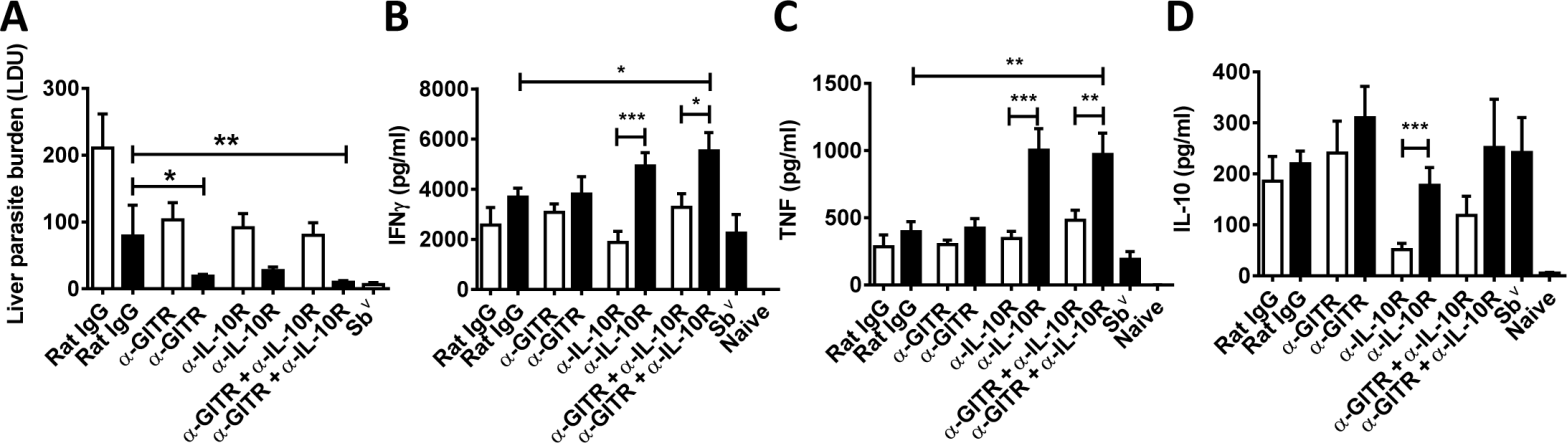
The effects of different immune and drug therapy combinations. *L*. *donovani*-infected mice (low-dose challenge with 5 x 10^6^ parasites) were treated either with control Ab, anti-GITR mAb, anti-IL-10R mAb or both mAbs with (black bars) or without (open bars) a sub-optimal dose of sodium stibogluconate (Sb^v^). A group receiving a full dose of Sb^v^ was also included. Infected mice were treated with anti-GITR mAb on day 14 p.i., and anti-IL-10R mAb on days 14, 19 and 24 p.i., with or without drug, as indicated. Rat IgG was used as a control. Liver parasite burdens were measured at day 28 p.i. (A). Spleen cells were also isolated at this time and cultured with parasite antigen for 24 hours before measuring levels of IFNγ (B), TNF(C) and IL-10 (D) in culture supernatants. Data are represented as the mean +/- SEM, and statistical differences of p < 0.05 (*), p < 0.01 (**) and p < 0.001 (***) are indicated (n = 6 mice per group, one independent experiment from 1–3 independent experiments).

Given that any immune therapy for a parasitic disease is likely to be combined with anti-parasitic drug treatment, we next examined the impact of combined anti-GITR and anti-IL-10R mAb treatment with a sub-optimal dose of the anti-parasitic drug sodium stibogluconate (Sb^v^). A low-dose infection was allowed to establish for 14 days before beginning drug treatment and/or antibody administration (Fig 6). Although drug reduced hepatic parasite burdens in all antibody treated groups, a significant improvement compared to mice treated with sub-optimal drug dose alone was only achieved when either anti-GITR mAb alone or combined anti-GITR and anti-IL-10R mAb were used (Fig 6A). We next assessed anti-parasitic cellular responses *ex vivo*. Spleen cells were used for these experiments because in our experience, cultured hepatic CD4^+^ T cells grow poorly, possibly reflecting a more advanced differentiated state that makes them more susceptible to death. When cellular responses from mice treated with antibodies combined with sub-optimal drug were compared to cell samples from mice treated with sub-optimal drug dose alone, significantly increased IFNγ and TNF production was only observed when combined with anti-GITR and anti-IL-10R mAb (Fig 6B and 6C). However, drug treatment with both combined anti-GITR and anti-IL-10R mAb, as well as anti-IL-10R mAb alone, resulted in significantly increased IFNγ and TNF production, compared to groups treated with the antibodies alone (Fig 6B and 6C). There was little difference in IL-10 levels, except in groups that received IL-10 signaling blockade alone, where IL-10 production was higher by cells from drug-treated mice. Together, these data indicate that combined antibody treatment together with anti-parasitic drug was effective at controlling parasite growth in the liver and promoting anti-parasitic immune responses.

**Fig 7:**
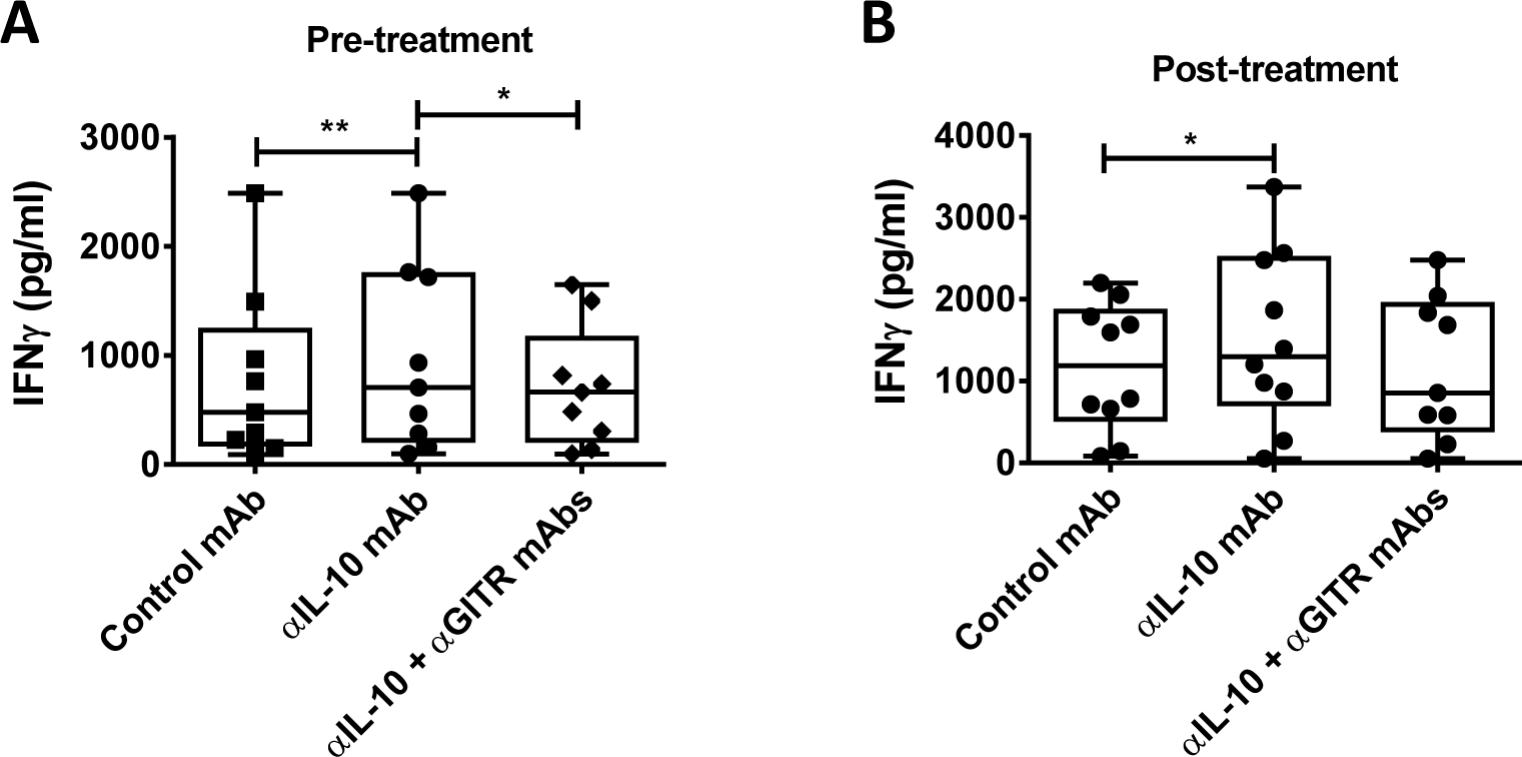
GITR activation alone or in combination with IL-10 blockade does not improve antigen-specific IFNγ production by whole blood cells after drug treatment. Antigen-specific IFN-γ production was measured on admission to clinic (**A**), and 24 hours after single-dose ambisome treatment (**B**), in whole blood cells cultured for 24 hours with a blocking anti-IL-10 mAb, with or without agonistic anti-GITR mAb, and compared with samples treated with control IgG1, as indicated (n = 10). Box and whisker plots show the box extending from the 25^th^ to 75^th^ percentiles with the line in the middle of the box representing the median and whiskers going down to the smallest value and up to the largest. Statistical differences of p < 0.05 (*) and p < 0.01 (**) are indicated.

Finally, we examined whether drug treatment affected the ability of combined mAb’s to influence cell-mediated immune responses in VL patients by again using IFNγ production in response to parasite antigen in a whole blood assay as a readout. Blood samples were taken from VL patients upon admission to clinic and 24 hours after drug treatment with a single-dose of liposomal amphotericin B[15]. Again, prior to treatment, anti-IL-10 mAb improved parasite-specific IFNγ production (Fig 7A), but when combined with anti-GITR mAb suppressed the enhanced IFNγ production following IL-10 blockade (Fig 7A). After drug treatment, IL-10 signaling blockade again improved parasite-specific IFNγ production (Fig 7B). However, at this time, despite no improvement in IFNγ production, there was no significant decrease in IFNγ levels by the addition of anti-GITR mAb to cells receiving IL-10 signaling blockade. Together, these results indicate that drug treatment reduced the negative impact of anti-GITR mAb on IL-10 signaling blockade, but anti-GITR mAb treatment is unlikely to offer significant therapeutic benefit to VL patients over IL-10 signaling blockade alone.

## Discussion

Our previous work identified GITR as a potential therapeutic target for treating VL[29]. Here, we show that not only did targeting GITR fail to improve anti-parasitic immune responses in VL patient samples, but had a negative impact on IL-10 signaling blockade, which alone can significantly improve parasite-specific T cell responses. Studies in an experimental model demonstrated that this negative effect was prominent in the liver, dependent on parasite burden and was associated with more Th1 cells expressing high levels of KLRG-1. Interestingly, this latter finding did not appear to indicate the promotion of T cell exhaustion because these KLRG-1^+^ Th1 cells produced high levels of IFNγ and displayed no increase in PD-1 expression. Instead, we speculate that the negative impact of anti-GITR mAb treatment on IL-10 signaling blockade was caused by either temporal changes in cytokine production by CD4^+^ T cells or the promotion of an unidentified immunoregulatory pathway. Nevertheless, combined IL-10 signaling blockade and anti-GITR mAb treatment could be improved when used with anti-parasitic drug. However, additional studies with VL patient samples indicated that targeting GITR had no advantage over IL-10 signaling blockade alone at improving anti-parasitic immune responses, even with drug treatment cover. Hence, our findings identify several important factors that influence the effectiveness of immune modulation, including parasite burden, target tissue and the use of anti-parasitic drug. Importantly, our results also highlight potential negative effects of combining different immune modulation strategies.

Results obtained from VL patient samples showed that despite increased expression of GITR by CD4^+^ T cells, anti-GITR mAb treatment failed to improve anti-parasitic immunity in a whole blood assay, and critically, reversed improvements in antigen-specific IFNγ production caused by IL-10 signaling blockade. Initial attempts to investigate this phenomenon in an experimental mouse infection failed to recapitulate these effects. However, when mice were infected with a lower parasite number, we found a similar negative effect of GITR activation on liver anti-parasitic immunity. It was surprising that a lower parasite inoculum resulted in more activated Th1 cells, compared with the frequency and numbers found in mice infected with a higher number of parasites. However, earlier studies by Parish and colleagues[46–48] described distinct patterns of cellular and humoral immune responses depending on antigen dose, with high dose of pathogen suppressing cell-mediated immunity. In fact, IL-10 levels are elevated in VL patients, and this cytokine suppressed parasite-specific CD4^+^ T cell IFNγ production, but after parasite burdens were reduced by drug treatment, IL-10 levels fell and parasite-specific CD4^+^ T cell IFNγ production increased[17,20,31]. Similar pathogen burden-dependent immunosuppressive mechanisms have been reported in other infections, including malaria[49,50] and hepatitis B[51]. Data presented in this study also showed a greater ratio of Th1 to Tr1 cells in control mice with low-dose infection, compared to the same group with a high dose infection, and this may contribute to a more activated status that results in a greater proportion of KLRG1^+^ cells.

These findings raise questions about which experimental settings provide the best reflection of clinical VL. The mouse model we have employed exhibits an acute, resolving infection in the liver, but not sterilizing immunity. In contrast, the spleen becomes chronically infected and maintains a relatively high parasite burden, associated with tissue pathology[52]. Hence, these two sites of infection in the mouse model appear to represent different ends of the human clinical VL spectrum. In recent years, VL patients generally present at clinics earlier during disease progression (Shyam Sundar, BHU, personal observations), possibly due to increasing awareness of disease and improved access to treatment via various programs aimed at VL elimination in the Indian subcontinent. Thus, it is possible that a low parasite burden setting in the liver of mice best reflects anti-parasitic immune responses in early clinical VL. However, this may not always be the case, and is likely to differ in situations where disease progression is either more rapid or advanced, such as might occur in different geographical locations where disease control programs are more limited[53].

Combining immune checkpoint inhibitors such as anti-CTLA4 and anti-PD1 mAbs can have dramatic effects on anti-tumor immune responses, and result in significant improvements in clinical outcomes, compared with single antibody treatment[54]. However, combining immune therapies does not always work. In a recent study, administration of agonistic anti-OX40 mAb to mice infected with *Plasmodium yoelii* resulted in significantly enhanced anti-parasitic T cell responses and improved control of parasite growth. However, when combined with PD-1 blockade, a strategy previously shown to improve anti-parasitic immunity during malaria[55], the beneficial effects of OX40 activation were reversed[56]. In this study, the combined therapy caused excessive T cell IFNγ production. Although we did not find enhanced Th1 responses with combined antibody treatment in our VL model, we did observe increased Th1 cell exhaustion, which was especially prominent in the liver when mice were infected with a lower parasite inoculum. Thus, it appeared that combined GITR activation and IL-10 blockade promoted excessive Th1 cell expansion, and subsequent exhaustion, without associated improvement in anti-parasitic immunity. The molecular mechanisms mediating this effect are unknown, but warrant further investigation if we wish to better understand and predict detrimental outcomes from immune modulation.

Importantly, when a sub-optimal drug treatment regime was incorporated into the combined anti-IL-10R and anti-GITR mAb treatment, significant improvements in anti-parasitic immunity were observed. Sub-optimal drug treatment worked most effectively with anti-GITR mAb alone or the combined anti-IL-10R and anti-GITR mAb treatment, relative to the control antibody treatment group. However, significantly improved IFNγ and TNF production in response to parasite antigen were as good in mice that received anti-IL-10R mAb alone, compared with mice receiving combined antibody treatment. We also examined the effect of drug administration on combined antibody treatment with VL patient samples by comparing responses to parasite antigen in whole blood assays 24 before and 24 hours after drug treatment with liposomal amphotericin B[14]. Although responses following combined anti-GITR and anti-IL-10 mAb administration after drug treatment were not significantly reduced, as was the case before drug treatment, there was no improvement in response, compared with IL-10 signaling blockade alone. One important difference between our studies in mice and humans was that pentavalent antimonials were used in mice, while liposomal amphotericin B was used in VL patients, and this may explain some differences we observed. We were unable to use amphotericin B in our studies because the efficacy of this drug against the LV9 strain (MHOM/ET/67/HU3) of *L*. *donovani* we use was poor, possibly reflecting the East African origins of this strain and the relatively poor efficacy of amphotericin B in VL patients in East Africa [57]. Nevertheless, based on the available data, GITR activation provided no additional advantage to anti-IL-10 mAb treatment alone at improving anti-parasitic immune responses within 24 hours of drug treatment. The effectiveness of the recently introduced, single-dose drug treatment protocol in VL patients in our study site means that patients are released after 24 hours after drug treatment, making sample collections at later times difficult, and changes in responsiveness to combined antibody treatment later during recovery harder to assess. However, given the substantial welfare advantages of the minimized hospital admission time with the new drug treatment protocol, any immune modulation aimed at improving anti-parasitic immunity would have to be administered at the time of drug treatment, thus reinforcing the superiority of anti-IL-10 signaling blockade alone.

Efforts to eliminate VL are likely to require coordinated efforts involving improved patient treatment, sustained vector control programs and strategies to reduce parasite loads in individuals to below levels that allow parasite transmission. A major risk factor for developing VL is that a household member has previously had this disease[58], thereby indicating that VL patients can act as parasite reservoirs for transmission. If transmission occurs after drug treatment, then the time of treatment might provide a window of opportunity to enhance immunity to reduce persisting parasite numbers. As described earlier, there have been many potential immune check point molecules identified that could be targeted and combined with drug treatment for beneficial outcomes. Furthermore, there is ample evidence for the establishment of regulatory mechanisms that suppress anti-parasitic immunity in VL patients[7]. Therefore, the development of immunomodulatory strategies, whether as single or combined treatments, has the potential to improve VL elimination programs. The challenge is to identify the right targets that achieve the above goals without causing harm.

In summary, our study shows that immune modulation can be an effective adjunct to parasite drug treatment. However, the effectiveness of such approaches will vary depending on clinical parameters such as parasite burden, and in the case of combined therapeutic approaches, whether the host response to individual components is complimentary or antagonistic. Therefore, targeting immune checkpoints has the potential to improve anti-parasitic immunity, but efforts to improve these responses by combining reagents to target different immune molecules should be evaluated carefully, taking into consideration the inclusion of conventional drug treatment approaches.

## Supporting Information

S1 Fig Combination antibody treatment results in increased KLRG-1 expression by Th1 cells in the spleen. **A.** Splenic CD4^+^ T cells from mice infected with a low (**B**) and high (**C**) parasite inoculum, and treated as indicated, were analysed by FACS by first gating on CD4^+^ and CD8^+^ populations in the TCRβ^+^ NK 1.1^-^ cell fraction. Activated CD4^+^ T cells were selected based on high levels of CD49d and CD11a expression. Intracellular cytokine staining on activated CD4^+^ T cells was used to identify Th1 (Tbet^+^ IFNy producing CD4^+^ T cells; measured *ex vivo*), Tr1 (IL-10 and IFNy producing CD4^+^ T cells; measured after 3 hours stimulation *ex vivo* with PMA and ionomycin) and terminally differentiated Th1 (Tbet^+^ KLRG-1^+^ CD4 T cells) cells. Infected mice were treated with the mAb alone or a combination of anti-GITR mAb on day 14 p.i., and anti-IL-10R mAb on days 14, 19 and 24 p.i., as indicated. Rat IgG was used as a control. Both the frequency and total number of cells are shown for each CD4^+^ T cell subset. Data are represented as the mean +/- SEM at day 28 p.i.. Statistical differences of p < 0.05 (*) and p < 0.001 (***) are indicated (n = 15 mice per group from 3 independent experiments).
(TIF)
