# Supplementary figures and images for: Combined Immune Therapy for the Treatment of Visceral Leishmaniasis
Source: PLoS Negl Trop Dis. 2016 Feb 12;10(2):e0004415. doi: 10.1371/journal.pntd.0004415 (PMC4752322; doi:10.1371/journal.pntd.0004415)

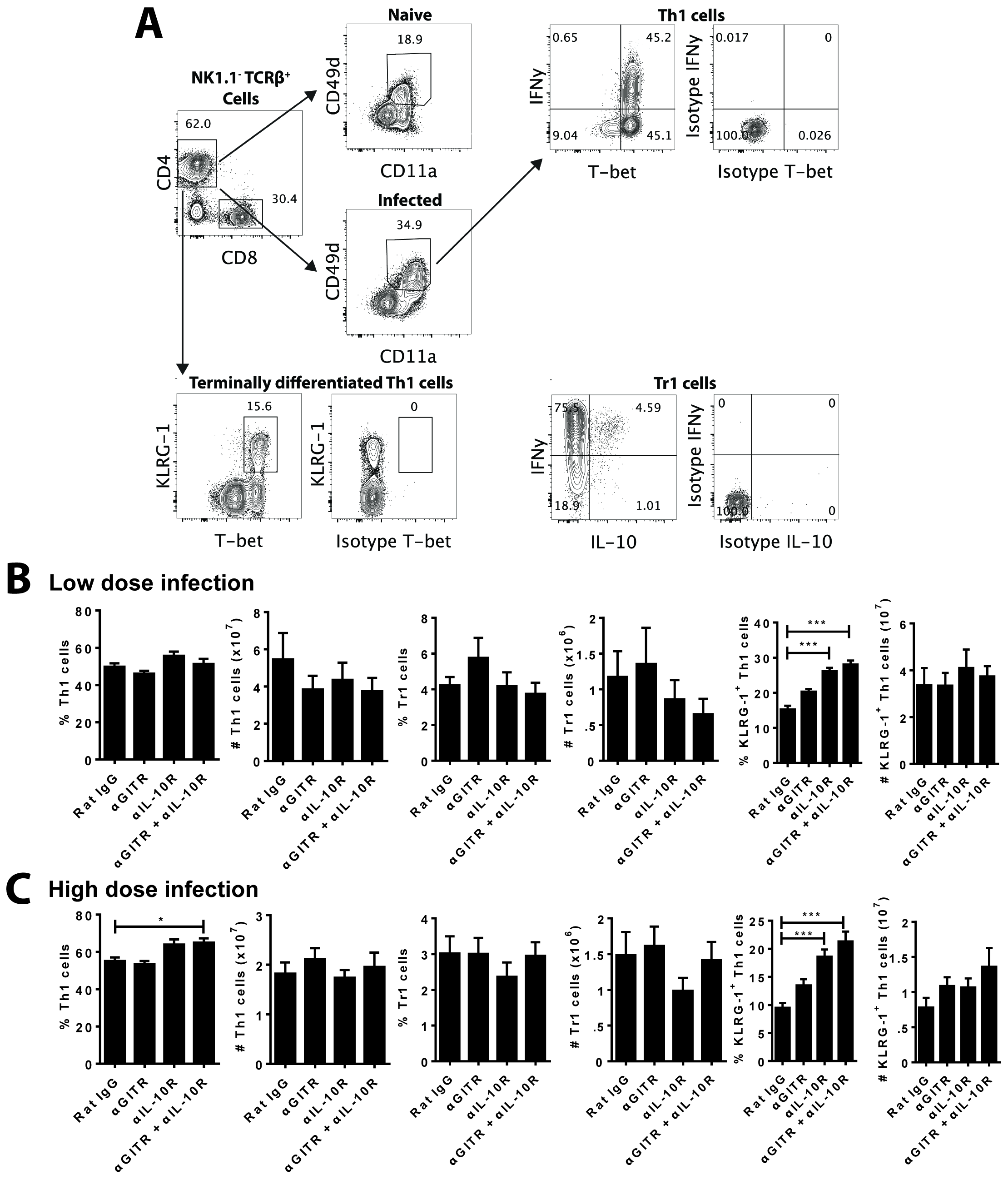

Supplement: S1 Fig — A. Splenic CD4+ T cells from mice infected with a low (B) and high (C) parasite inoculum, and treated as indicated, were analysed by FACS by first gating on CD4+ and CD8+ populations in the TCRβ+ NK 1.1- cell fraction. Activated CD4+ T cells were selected based on high levels of CD49d and CD11a expression. Intracellular cytokine staining on activated CD4+ T cells was used to identify Th1 (Tbet+ IFNy producing CD4+ T cells; measured ex vivo), Tr1 (IL-10 and IFNy producing CD4+ T cells; measured after 3 hours stimulation ex vivo with PMA and ionomycin) and terminally differentiated Th1 (Tbet+ KLRG-1+ CD4 T cells) cells. Infected mice were treated with the mAb alone or a combination of anti-GITR mAb on day 14 p.i., and anti-IL-10R mAb on days 14, 19 and 24 p.i., as indicated. Rat IgG was used as a control. Both the frequency and total number of cells are shown for each CD4+ T cell subset. Data are represented as the mean +/- SEM at day 28 p.i.. Statistical differences of p < 0.05 (*) and p < 0.001 (***) are indicated (n = 15 mice per group from 3 independent experiments). (TIF) [file pntd.0004415.s001.tif]
